# Does time pressure reveal habits? Divergent results from outcome devaluation and response remapping paradigms

**DOI:** 10.3758/s13415-026-01474-6

**Published:** 2026-08-05

**Authors:** Parnian Rafei, Steph F. Suddell, Eike K. Buabang, Gonzalo P. Urcelay, Claire M. Gillan

**Affiliations:** 1 Trinity College Institute of Neuroscience (TCIN), Trinity College Dublin, Dublin, Ireland; 2 School of Psychology, Trinity College Dublin, Dublin, Ireland; 3 School of Psychology, University of Nottingham, Nottingham, UK

**Keywords:** Habits, Goal-directed control, Outcome devaluation, Response remapping, Action slip, Stimulus-response associations, Instrumental learning

## Abstract

Habits are central to daily life but remain difficult to study in the lab. Outcome Devaluation is often considered a “gold standard” paradigm for habit measurement; yet human studies have produced mixed evidence: many fail to show the overtraining effects on devaluation sensitivity expected from a robust habit index. An alternative measure involves remapping stimuli to new responses rather than devaluing outcomes. Crucially, this procedure limits preparation time to reveal habits that may otherwise be masked by goal-directed processes. Although promising, it remains unclear whether these tasks assess the same behavioural phenomenon. To address this, we compared Outcome Devaluation and Response Remapping under matched preparation-time constraints after 6 days of training. Response Remapping showed the expected pattern: expression of the pretrained (putatively “habitual”) response was greatest at 200–500 ms and weakened as preparation time increased. In Outcome Devaluation, raw responses to devalued outcomes increased with preparation time, driven by a time-dependent withholding bias. Once controlled for, devalued responses decreased with preparation time. Crucially, this effect was larger in Remapping. Moreover, task comprehension problems may limit interpretation of the Devaluation task. These findings suggest that paradigms engage partially distinct processes—response inhibition versus automatic action selection—with one requiring inhibition of a learned response and the other relying on fast, automatic actions. Our findings reveal that behaviour on these tasks is sensitive to task structure, underscore the need to consider distinct cognitive and motivational factors driving action selection, and highlight that what researchers call “habits” is influenced by multiple processes.

**Supplementary Information:**

The online version contains supplementary material available at https://doi.org/10.3758/s13415-026-01474-6.

## Introduction

Understanding how habits form and manifest in human behaviour remains a key question in psychology and neuroscience. While the definition of habit varies across disciplines (Handel & Smith, 2024), habits in cognitive neuroscience are commonly defined as automatic behaviours that are triggered by stimuli rather than being driven by a representation of a current goal (Buabang et al., 2025a, 2025b; Dickinson, 1985). The foundation of habits are stimulus–response (S-R) associations, which develop through repetition. However, habit expression is more complex than that and can be constrained by the goal-directed processes, for example, the extent to which response-outcome (R-O) associations or outcome values are considered at the time of choice (Balleine & O’Doherty, 2010). The expression of habit is therefore governed by a balance between S-R and goal-directed systems. This balance is crucial for everyday functioning and is thought to be impaired in psychiatric disorders, where maladaptive habits persist despite negative consequences (Verhoeven & de Wit, 2018). Specifically, excessive habit expression has been implicated in compulsive disorders, such as obsessive–compulsive disorder (OCD) (Gillan et al., 2011; Vaghi et al., 2017) and various types of addiction (Corbit et al., 2014; Everitt & Robbins, 2016; Voon et al., 2015, but see Buabang et al., 2025a, 2025b; Hogarth, 2020).

Despite considerable interest, studying habits in the lab has proven difficult. Outcome Devaluation, the gold-standard paradigm, operationalises habits as previously trained behaviours that persist even when the associated outcome loses value (Balleine & Dickinson, 1998; Pierce-Messick et al., 2025). In this method, participants learn to associate stimuli with responses that lead to rewarding outcomes. These outcomes are then devalued—either through explicit instructions (e.g., penalties in the slips-of-action task; Watson et al., 2018) or reduced motivational appeal (e.g., satiation or taste aversion; Buabang et al., 2023a, 2023b; Tricomi et al., 2009). Studies in rodents have used this paradigm to dissociate the brain systems involved in S-R learning, goal-directed control, and habit expression, and examine the conditions that promote or curtail habits (Colwill & Rescorla, 1985; Handel & Smith, 2024).

In humans, however, the Outcome Devaluation test has been less successful. For example, studies have consistently failed to find evidence for one of the perhaps most fundamental principles of habit—that the likelihood of behaving habitually increases following extensive training of S–R associations (de Wit et al., 2018; Gera et al., 2023; Pool et al., 2022). A common challenge for devaluation that has been highlighted in the literature is that the procedure is commonly subject to misinterpretation by participants, affecting its validity (Buabang et al., 2021; De Houwer et al., 2018). Another reason for these inconsistencies is that “habit expression” as operationalised in the Outcome Devaluation task can arise via two routes: either decreases in goal-directed control or increases in S–R associations (Watson & de Wit, 2018). Although Outcome Devaluation cannot disambiguate these processes, when deployed in humans, some have argued that habit expression may be mostly driven by variation in goal-directed processes (de Wit et al., 2018; Luque et al., 2020). If this is the case and goal-directed control is the main driver, then overtraining S–R associations alone will not reliably increase measured “habit” unless goal-directed control is simultaneously taxed, meaning that changes in S–R strength would remain difficult to detect without a manipulation that impairs or burdens the goal-directed system.

One way that this might be achieved is by examining behaviour under time pressure. Different pieces of evidence suggest that the stimulus-driven system operates faster than goal-directed control (Keramati et al., 2011). We therefore might be able to measure S-R processes without interference from the goal-directed system by limiting the time people have to select a response. Testing this idea, Hardwick & colleagues (2019) found evidence that overtraining boosts habit expression following 4 and 20 days of training but only on trials where preparation time was limited (Hardwick et al., 2019). Instead of using Outcome Devaluation to probe habits, in this task, participants are required to learn associations between stimuli and responses and then revise these associations through instructions (termed “Remapping”). They are then tested for the expression of old S-R links across a range of trials with varied amounts of preparation time. Preparation time in this study is manipulated by requiring participants to always respond in tandem with the fourth of four equally spaced tones, while randomising when the stimulus appears relative to that final tone. While the key finding from Hardwick & colleagues’ (2019) Remapping task has been replicated several times (Buabang et al., 2023a, 2023b; Du & Haith, 2025; Martínez-López et al., 2025; Van Dessel et al., 2024)—that habits are strong on trials with short but not long preparation times—it is unclear whether the effect of overtraining on behaviour is evident because of the introduction of a time pressure manipulation or because the critical test is a Remapping task that may not require more traditional goal-directed processes (e.g., outcome valuation, prospective planning) and has no overt relationship to reward. In other words, it is presently unclear whether time constraints alone can reveal the impact of training duration on habit expression following Outcome Devaluation.

One reason that we might question the convergent validity of these operationalisations of habit comes from a recent study comparing habit slips on the Remapping task to a potentially related metric: response switching reaction time cost (Martínez-López et al., 2025). This switch cost measure was developed to serve as a more sensitive measure than commission errors, frequently (but unsuccessfully) used in the literature (Luque et al., 2020). Notably, when these metrics were tested in the same participants, there was no correlation between the critical indexes of habits in the two paradigms. Another recent study examined outcome *re*-valuation (note that as opposed to devaluation, revaluation involves both increases and decreases in value) under time pressure after a single day of training. They found only tentative evidence for more habit expression in trials with short versus long preparation time (in younger adults only) (Hennessy et al., 2025). Crucially, habit effects were only observed on “up-valued” trials (necessitating a *go* response) and not trials where the outcome was devalued (necessitating a *no-go* response). One possibility is that this lack of effect is due to the short duration of training employed. Indeed, another study compared response withholding to switching (both under time pressure) also following one day of training and found much stronger evidence for habits with the latter, suggesting that Remapping and withholding may probe distinct aspects of habit: response preparation (i.e., selecting an action) versus response initiation (i.e., executing the action (Du & Haith, 2025)). Interestingly, they found that they could show evidence for over-training effects on withholding (i.e., commission errors), but only when they used pre-learned associations acquired outside the lab (i.e., alphanumeric mappings A-1, B-2). These findings highlight the complexity of habit expression and suggest that different paradigms may tap into distinct facets of habitual behaviour (Nebe et al., 2024), which may require different durations of training.

**Fig. 1:**
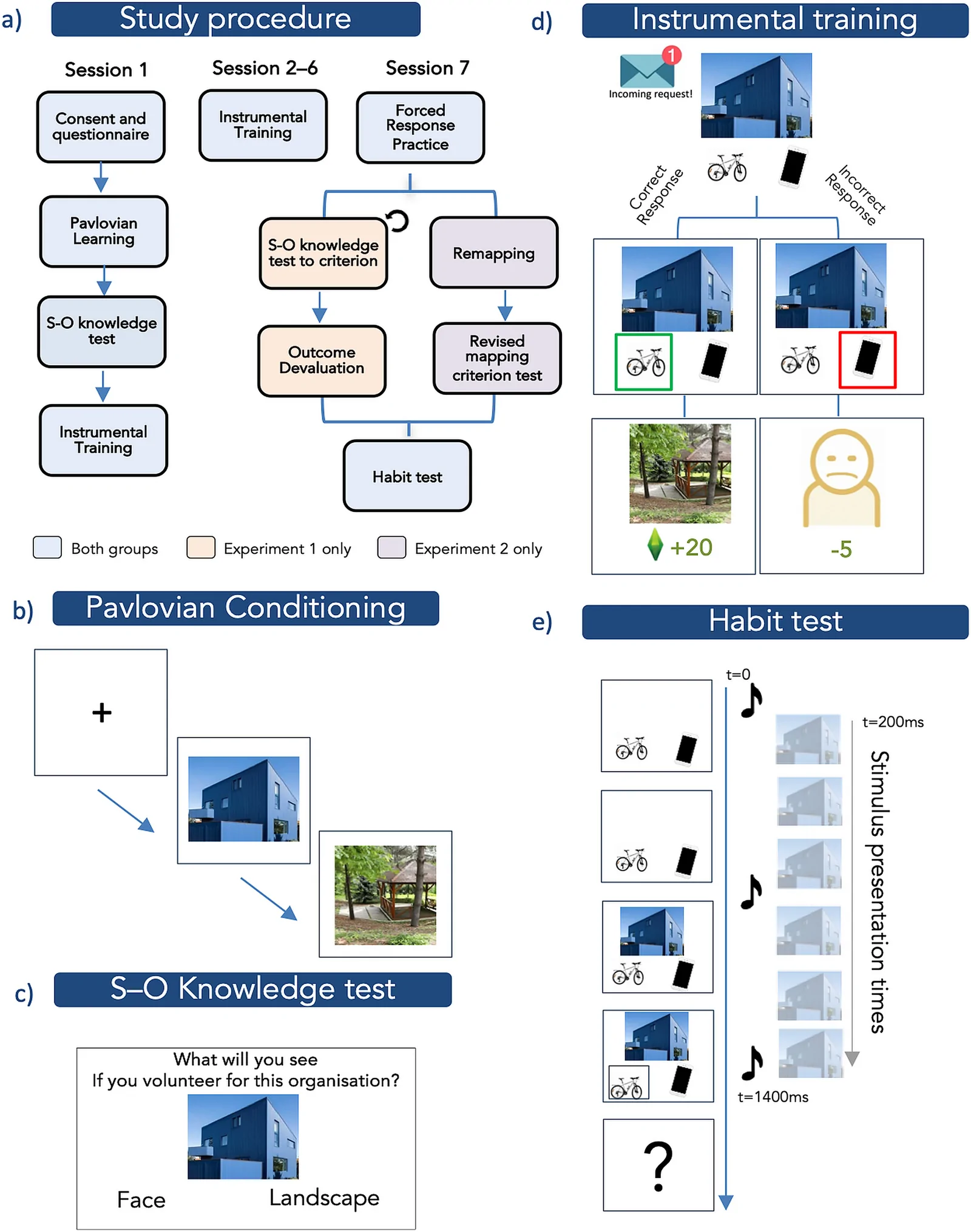
Paradigms and procedures. (**a**) The overall procedure for Experiments 1 (Outcome Devaluation) and 2 (Remapping) with colour coding the shared and unique elements of the procedure (**b**) A Pavlovian conditioning phase took place at the beginning of the first session, comprising 20 trials requiring passive viewing of four sets of novel associations between stimuli (pictures of yellow, blue, red or green buildings) and outcomes (pictures of landscapes or faces). (**c**) S–O explicit knowledge test, in which participants were tested for their awareness of the outcome related to each stimulus. (**d**) The sequence of instrumental training trials (correct and incorrect responses), spaced evenly over 6 days and required participants to respond to stimuli (pictures of yellow, blue, red or green buildings) with one of two responses (phone or bike), leading to point gain (+ 20) upon each correct or point loss (− 5) for each incorrect response (**e**) The habit test trial during the test session (both experiments). A forced-response reaction time paradigm required participants to respond when the third tone was played. Responses were shown from the beginning of each trial, but stimulus onset times varied to control the amount of response preparation time (between 0.2–1.4 s) on each trial. Stimulus onset times were drawn from a uniform distribution, and trials where the stimulus appeared earlier had more preparation time. To prevent new learning during the test, participants were shown a question mark, instead of the outcome (i.e., pictures of faces or landscapes)

The present study aimed to bridge an important gap in this literature and directly test whether time constraints reveal habitual responding following Outcome Devaluation of a well-trained behaviour. Conceptually, Outcome Devaluation and response Remapping both assay “habitual slips,” but they place demands on different aspects of cognitive control. In Outcome Devaluation, action selection is guided by S–O–R knowledge; “habit expression” appears when participants fail to withhold a previously reinforced response because the expected outcome is now devalued (i.e., a failure of outcome-based inhibition). In response Remapping, participants must rapidly re-select an instructed S–R mapping under time pressure, and “habit expression” appears when the old S–R wins the race against the updated rule. Thus, devaluation hinges on expected consequences and inhibition, whereas Remapping hinges on fast S–R capture versus reselection. Unlike prior studies, we directly compared performance on Outcome Devaluation (Experiment 1) to a version of the Remapping task introduced by Hardwick & colleagues (2019) (Experiment 2) (Hardwick et al., 2019) that was specifically designed to have an analogous training phase (Fig. 1). This means that after 6 days of comparable S–R-O training, we could directly compare what is defined as ‘habit expression’ in the two tasks: i.e., sensitivity to outcome devaluation and accuracy in remapping a pre-trained response to a new stimulus. Our goal was not to claim that Outcome Devaluation and response Remapping index entirely distinct “types” of habit, but to test whether, under identical training and timing constraints, these paradigms yield different behavioural signatures of habit expression. The two tasks operationalise control differently (withholding a response to a devalued outcome vs rapidly re-selecting an updated S–R), so apparent differences could arise either from “mapping” differences or from the response mode required. Crucially, both tasks used the same preparation time constraint in the test phase, with continuously varying preparation times (0.2–1.4 s), allowing us to systematically examine any differences in the time dependence of habitual responding across the paradigms. We hypothesised that shorter response preparation times would increase habitual expression in both tasks and aimed to empirically test the common suggestion that the expression of habitual action slips in devaluation tasks may have been masked by allowing participants ample time to prepare their response in a goal-directed manner.

## Methods

### Experiment 1 – Outcome devaluation task

#### Participants

A total of 42 participants were recruited via Prolific (https://www.prolific.co/). The inclusion criteria were 1) aged 18 to 60 years; 2) based in the UK or Ireland; 3) English language fluency; 4) normal or corrected-to-normal vision; 5) no colour-blindness; and 6) previous participation in at least 20 studies on Prolific. Twelve participants missed at least one training session and were therefore exited from the study, and one completed all training sessions but did not meet the inclusion criterion of 75% accuracy or above on the final training session. Thus, the final sample was 29 participants (M = 35.57 years, *SD* = 6.6 years) (53.6% women, 75% White, 64.3% undergraduate degree level; see supplementary Table 1 for full demographic information). All participants gave written informed consent, and all procedures were approved by the Trinity College Dublin School of Psychology Institutional Review Board. Participants received financial compensation (£7 per hour and a maximum of £4 bonus payment depending on their performance).

#### Design and procedure

This study consisted of seven sessions: six training sessions that were required to take place within a 10-day period and one test session at the end (Fig. 1a). The full description of additional procedures is available in the supplementary materials methods section. Upon signing up, participants were provided with a comprehensive digital information sheet, receiving general information about the study, and provided with an informed consent form. All the questionnaires and tasks were hosted by Pavlovia (https://pavlovia.org/) and built in PsychoPy (version 2024.1.4) (Peirce, 2007).

#### Behavioural training

After providing written consent and self-report data, participants were introduced to a task narrative. They were told they were playing a game as “Sam”, an avatar. Sam liked meeting people and getting out in nature, and Sam would achieve these goals if they volunteered for organisations—depicted as four coloured buildings (i.e., yellow, blue, green, and red). In the first phase of the task, Sam learned which of these organisations (stimuli) were linked to seeing people or getting out in nature (outcomes – pictures of natural landscapes or faces) in a Pavlovian conditioning phase. There were 20 trials in total; each of the four stimuli was presented five times, immediately followed by its associated outcome from the two categories (i.e. face or landscape; Fig. 1b). Two of the organisations, the stimulus–outcome associations, were counterbalanced between participants. After finishing the Pavlovian conditioning task, participants completed an explicit knowledge test of the stimulus-outcome associations that they had just learned (Fig. 1c). The test consisted of eight trials—two for each association. The participants received feedback about their response accuracy after each trial. Data from two participants were not recorded for this test owing to technical issues. For the purpose of another study for which this dataset will serve as a control group, participants next completed an unrelated mental simulation task (described in the supplementary materials).

Next, participants completed instrumental training, where their avatar, Sam, learned through experience which response they should perform when contacted by each organisation. There were two options: “calling them on a phone” and “cycling to the office,” shown at the bottom of the stimulus on the same side for each trial. If they made the correct response to a given stimulus within 1500 ms, Sam was rewarded with the corresponding outcome for that stimulus (nature or faces) and received “happiness points” (Fig. 1d). Participants were instructed that making a response was effortful and, therefore, cost 5 happiness points (response cost), but each correct response resulted in gaining 20 happiness points; hence, the participants would lose 5 points for each incorrect response. The participants first completed 60 practice trials in session one without gaining or losing any points. Each training session thereafter (sessions 1–6) consisted of 240 reinforcement learning trials (60 per organisation–i.e., stimulus), resulting in 1440 training trials for each participant across 6 sessions.

#### Habit test: Outcome devaluation

Following training, we conducted an Outcome Devaluation test under time pressure, with the goal of assessing whether participants would continue to initiate the practised responses for these previously-valued outcomes (i.e., action slips), or whether they could flexibly withhold them. Participants were informed that “Sam” is now bored of one of the two outcomes from training (being in nature or meeting people, counterbalanced), and to avoid losing points, they need to withhold their responses to the organisations linked to this “devalued outcome”. The other outcome was still valued; hence, the participants had to withhold their responses for the two organisations linked to the devalued outcome and actively respond to the other two still-valued ones. Based on the findings of previous studies (Hardwick et al., 2019), we expected that the habitual system can select potential responses rapidly, while the goal-directed system requires longer processing times for withholding responses to devalued outcomes. We, therefore, imposed varying degrees of time pressure on this Outcome Devaluation test. Three tones were played during each trial of the test, and participants were required to respond at the same time as the third tone. The third beep tone (i.e., the allowed response time window) was played 1.4 s after the trial onset. Critically, the stimulus appeared at a random time between 0.2 to 1.4 s since the beginning of the trial (Fig. 1e). When the organisation was presented early, participants, in theory, had ample time to prepare the correct response, but when it was presented late, prior research has shown that habits are expressed (Hardwick et al., 2019). Prior to learning that outcomes were devalued and completing the test, participants completed a practice session with the time pressure manipulation and had to achieve 80% on-time responses before moving forward (for results, see supplementary materials, Fig. S5a). Before the final test, they also repeated the explicit knowledge test from day 1 to measure outcome knowledge and, in those who made errors, to provide feedback and ensure a basic level of awareness of stimulus-outcome relationships needed for the devaluation test. More details on these phases are available in the online supplement. To prevent new learning during the devaluation test under time pressure, participants were shown a question mark, instead of the outcome (i.e., faces or landscapes). If participants did not respond on time, they did not receive feedback about their timing and moved on to the next trial. The Outcome Devaluation test consisted of 360 trials (18 blocks of 20 trials), allowing the participants a response preparation time between 0.2–1.4 s, which was randomly distributed across trials.

### Experiment 2 – Remapping task

#### Participants

For experiment 2, we recruited 34 participants from Prolific with the same inclusion and exclusion criteria. Six participants dropped out because of missing one or more training sessions or failing to meet the inclusion criterion of 75% accuracy or above on the final training session. Thus, the final sample for experiment 2 consisted of 28 participants (*M* = 37.9 years, *SD* = 10.7) (56.7% women, 40% men, 3.3% nonbinary, 76.7% White, 36.7% undergraduate degree level, see Supplementary Table 1 for full demographic information).

#### Design and procedure

Experiment 2 had the exact same design and procedures during the training sessions (sessions 1–6; Fig. 1a) and only used a different paradigm in the test session for measuring habitual action slips (Hardwick et al., 2019).

#### Habit test: Response reversal

During the test session on the seventh day, participants learned that two of the organisations had changed their preferred method of communication, while the other two remained the same. Therefore, they needed to learn the revised S–R mapping to be able to respond to them accurately, with the goal of assessing whether participants would habitually prepare the previously practised response or could flexibly act according to the revised mapping. After being introduced to the forced-response task and completing criterion test block(s) for reaching 80% of on-time responses (for results, see supplementary materials), participants received instructions about the revised mappings. Following the instructions, they had to learn this revised mapping through a practice block consisting of 24 trials (6 per stimulus). The participants could only proceed to the main test blocks if they had responded to > 80% of the revised mapping practice trials accurately; otherwise, they had to repeat the block. They were instructed at the beginning that there was no time pressure in this practice block and that they should focus on learning a new S–R mapping regardless of time constraints. More details on task procedures are available in the online supplement. As in Experiment 1, the main blocks of the test session required participants to respond under time pressure (0.2–1.4 s), they were shown a question mark instead of the outcome (i.e., faces or landscapes, Fig. 1e) and were not given feedback on the timing of their response, and moved on to the next trial immediately (18 blocks of 20 trials; 360 trials in total).

#### Data analysis

All statistical analyses were carried out using the R programming language (R Core Team, 2024, version 4.4.1). We ran linear mixed-effects models with the *lmer* function from the *lme4* and ANOVAs with the *lmerTest* package in R (Bates et al., 2015; Kuznetsova et al., 2017) to test the training effects (i.e., effects of session on response reaction times and accuracy), and effects of preparation time on different response conditions (i.e., correct, incorrect, habitual, withheld). This analysis approach aimed to increase the precision of estimated group (fixed) effects by explicitly modelling the variance of the dependent variable associated with specific participants or training sessions (random effects). Pairwise comparisons between preparation time bins were conducted by using Tukey-adjusted post-hoc tests with the *emmeans* package (Lenth, 2024). In the Outcome Devaluation task, habitual responses were defined as making the previously trained response to stimuli that were associated with devalued outcomes. In the response reversal (henceforth referred to as the *Remapping* task), a habitual response was defined as making the previously trained responses on trials where the response was reversed. Only responses that were executed within the allowed time window (0.1 ± 0.1 s for the duration of the third beep tone) were considered valid and were added to the analysis, although analyses with a more lenient threshold are provided in the supplement for the interested reader (see Fig. S3 for results without considering time constraints). For the primary analyses, we used 300-ms time bins spanning 0.2–1.4 s to match our tasks’ forced-response schedule; however, because prior Remapping work highlights ~ 0.3–0.6 s as a critical early window for habits to unfold (Hardwick et al., 2019), we repeated all models with 0.3–0.6 s as the reference bin as a sensitivity analysis (full results in Supplement, Table S2a-e). A significance level of *p* < 0.05 was used for all statistical comparisons.

The sample size was based on Cohen’s *d* = 1.0 for between-task comparisons, taken from a prior study (Du & Haith, 2025). This required a minimum of 17 participants per group to achieve the 80% power. To be conservative, we oversampled somewhat in each group, with some uncertainty about retention and exclusions over the longitudinal study period. The institutional review board ethical approval was obtained from the Research Ethics Committee of the School of Psychology, Trinity College Dublin (Approval ID: SPREC012022-14). All research was performed in accordance with relevant guidelines and regulations, including those outlined in the Declaration of Helsinki. Both experiments only included adults older than 18, and informed consent was obtained from all participants.

## Results

### Instrumental training: Reaction times and accuracy and trials to criterion

**Fig. 2:**
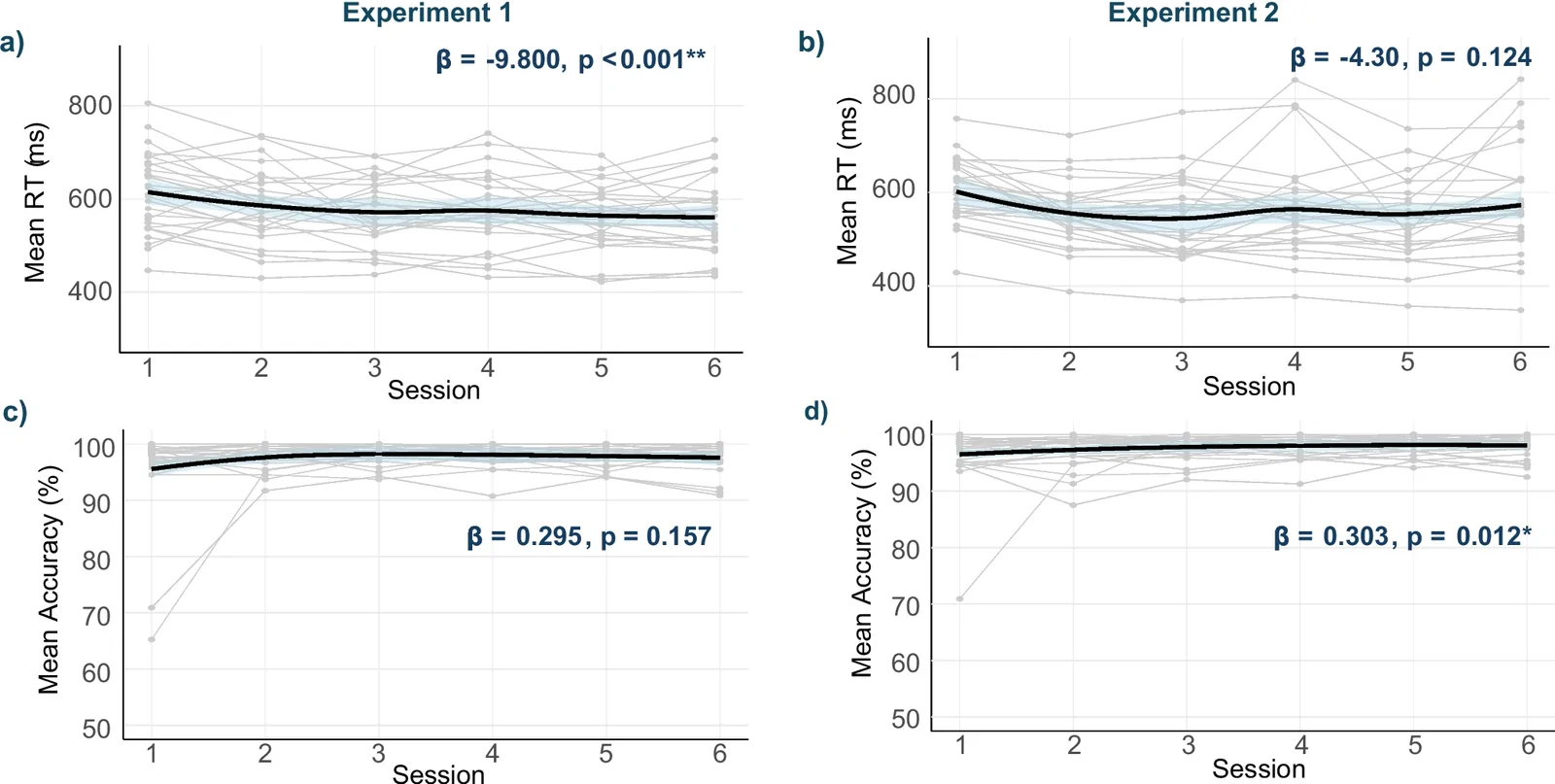
Training effects on response reaction times and accuracy across sessions. (**a**) Response reaction time (RT) changes across sessions for the outcome devaluation test group. (**b**) Response reaction time changes across sessions for the remapping test group. (**c**) Response accuracy changes across sessions for the outcome devaluation test group. (**d**) Response accuracy changes across sessions for the remapping group

We first ran linear mixed-effects models to examine the change in reaction times (RT) across the six training sessions, which were identical across the two experiments. In the Outcome Devaluation task, we observed a significant decrease in RT across sessions (β = − 9.80, SE = 2.393, *p* = 0.0003) (Fig. 2a). In the Remapping task, we observed the same pattern for the change in reaction time across sessions—a slight decrease in RT (Fig. 2b). However, the change was not statistically significant (β = − 4.309, SE = 2.73, *p* = 0.124), likely due to a couple of outliers in the remapped group who slowed down considerably in the final days. We checked for outliers and flagged ID × session RTs as outliers when they deviated by more than 3 SDs from the session mean; this identified three participants (sessions 4 and 6). We re-ran the model excluding those session outliers and found that this recovered the expected RT improvement effect over training sessions (β = − 6.67, SE = 2.35, *p* = 0.0086). When comparing the two tasks, there was no main effect of task in the first session (β = − 33.9, SE = 18.94, *p* = 0.07), although there was a trend toward overall faster responding in the Remapping version. There was no interaction between session and task (β = 5.49, SE = 3.658, *p* = 0.139).

Next, we examined accuracy (i.e., the number of correct responses to training trials) across all training sessions in the same manner. In the Outcome Devaluation task, the effect of session on accuracy was not statistically significant (β = 0.30, *SE* = 0.203, *p* = 0.157) (Fig. 2c). The average accuracy across sessions for the participants in the Outcome Devaluation group was 96.4% (*SD* = 18.6). In the Remapping task, the average accuracy was similarly very high at 96.7% (*SD* = 17.7), but there was a statistically significant improvement in accuracy over sessions (β = 0.303, *SE* = 0.115, *p* = 0.012; Fig. 2d). Similar to the RT analyses, the interaction (session × task) showed no difference between the tasks, β = − 0.056, *SE* = 0.268, *p* = 0.835).

Finally, in the Outcome Devaluation task, the number of blocks the participants needed to reach 80% of on-time responses ranged between 1–5 (mean (*SD*) = 1.76 (0.78)). In the Remapping task, the number of blocks needed ranged between 1–6 (mean (*SD*) = 1.86 (1.27); Fig. S5a). A two-sample *t*-test revealed no significant difference between the two tasks in terms of the number of blocks to reach the 80% threshold (*t* (46.62) = 0.372, *p* = 0.711). Thus, neither reaction times nor each of the two indicators of accuracy differed across the groups; again, this is expected as the tasks were identical up until this point.

### Outcome devaluation test results

**Fig. 3:**
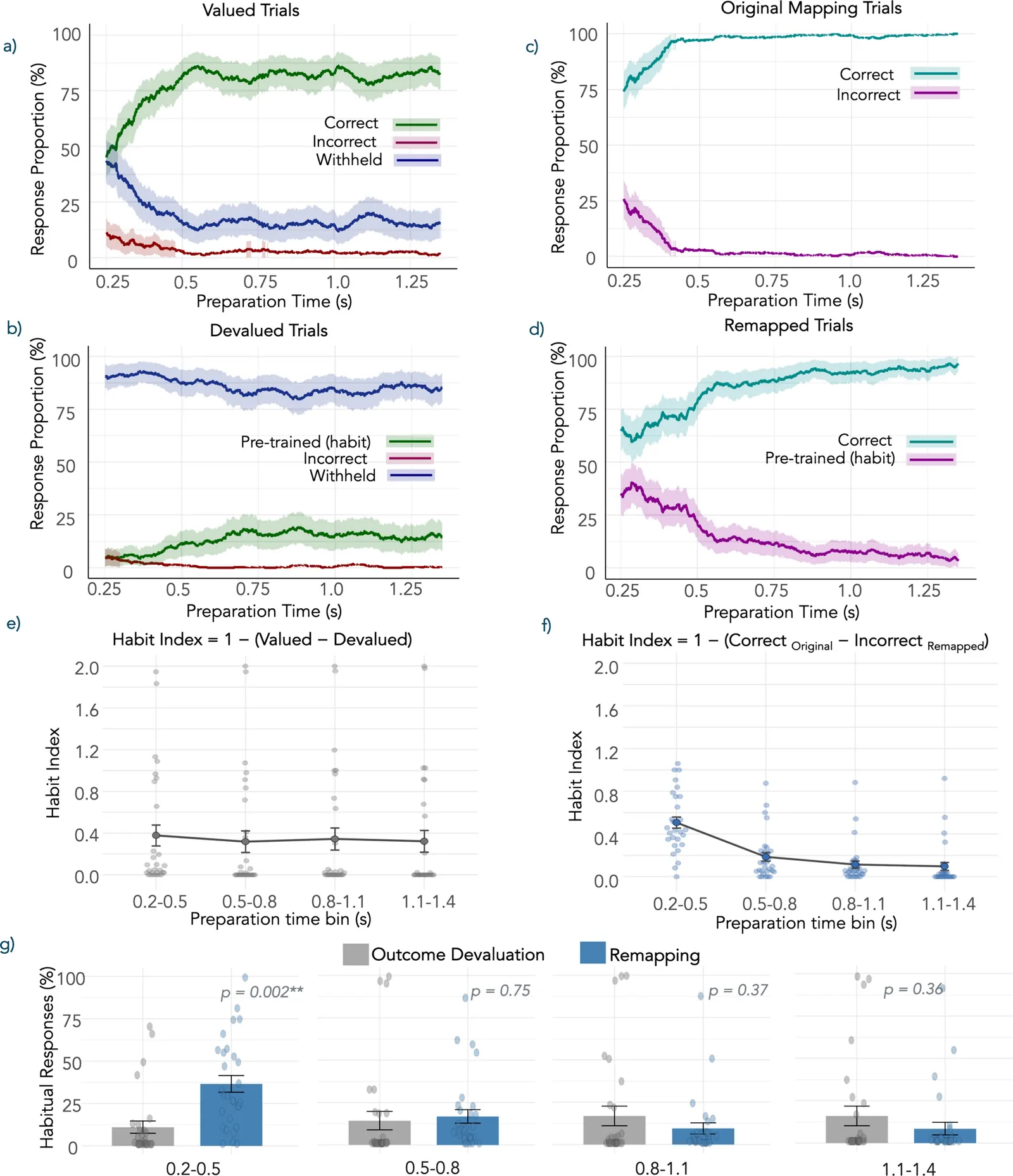
Response patterns as a function of preparation time in the outcome devaluation and remapping tests. (**a, b)** On-time only proportions (%) of correct, incorrect, and withheld responses for (**a**) still-valued and (**b**) devalued trials in the outcome devaluation test. (**c, d**) On-time only proportions (%) of correct, incorrect, and habitual (i.e., pre-trained) responses for (**c**) original consistent mapping and (**d**) remapped trials in the remapping test. (**e**) Outcome devaluation Habit Index, defined as *1 − (P[press|Valued] − P[press|Devalued])* (f) Remapping Habit Index, defined as *1 − (P[correct press|Original mapping] − P[incorrect press|Remapped])*. (**g**) The average proportion (%) of habitual responses across time bins and between tasks during the devalued and remapped trials, where we expected habits to emerge. Lines show participant means; shaded ribbons indicate ± SE. The error bars represent standard errors. Data reflect only on-time responses, calculated using a sliding-window analysis with 100-ms bins (first point at 250 ms, corresponding to the 200–300-ms bin). For complementary analyses, including all responses irrespective of timing for panels **a–d**, see Supplementary Fig. S3

In the Outcome Devaluation task, participants were informed that one of the outcomes was no longer valuable (seeing people or visiting landscapes) prior to the test phase. We then tested if they would habitually initiate the previously trained responses for stimuli associated with this outcome or could flexibly withhold them. We used a forced-reaction-time approach to test if this might be more apparent on trials with short preparation times (Fig. 1b). We ran a linear mixed-effects model to examine the effect of preparation time bins on habitual responses, i.e., (formerly) correct responses to devalued trials (*correct* ~ *binned response preparation time* + *(1 | ID)*). We set the first time bin (0.2–0.5 s) as the reference in our model, comparing all other time windows to this. Directly contrary to expectations, habitual responses increased, rather than decreased, with more preparation time. Although the increase from 0.2–0.5 to 0.5–0.8 s was only marginal (β = 3.81, *SE* = 1.96, *p* = 0.056), the effect became significant in the 0.8–1.1 s (β = 6.56, *SE* = 1.96, *p* = 0.001) and 1.1–1.4 s windows (β = 5.57, *SE* = 1.96, *p* = 0.006; Fig. 3b). However, after excluding the two participants who were doing the opposite of the behaviour commensurate with the devaluation instructions, the contrast from 0.2–0.5 s to 0.5–0.8 s was not significant (β = 0.66, SE = 1.44, p = 0.65); the 0.8–1.1 s contrast remained significant (β = 3.41, SE = 1.44, p = 0.020); and the 1.1–1.4 s contrast became non-significant (β = 2.37, SE = 1.44, p = 0.103; see supplementary results, Figure S7).

Next, we tested whether these habitual responses were more numerous than other errors. We calculated a difference score between the proportion of previously trained responses (i.e., correct) and the proportion of never-trained (i.e., incorrect) responses within each time bin (*(correct – incorrect)* ~ *binned response preparation time* + *(1 | ID*)). Overall, the habitual responses were not different from incorrect responses in the reference time bin (0.2–0.5 s) (β = 5.91, SE = 5.32, *p* = 0.275), suggesting no clear dominance of habitual over incorrect responses in the earliest preparation window. However, significantly more habitual than incorrect responses emerged as preparation time increased. In the 0.5–0.8-s time bin, habitual responses significantly outnumbered incorrect ones (β = 9.06, SE = 3.11, *p* = 0.005). This trend continued into later time bins, with stronger evidence in the 0.8–1.1-s (β = 11.18, SE = 3.11,* p* = 0.0004) and 1.1–1.4-s time windows (β = 10.65, SE = 3.11, *p* = 0.001). The same results held even after excluding the 2 participants who were not clearly following the devaluation instructions (0.2–0.5 s reference bin β = 4.47, SE = 3.62, p = 0.23; 0.5–0.8 s β = 3.54, SE = 1.80, p = 0.053; 0.8–1.1 s β = 5.64, SE = 1.80, p = 0.002; 1.1–1.4 s β = 4.94, SE = 1.80, p = 0.008). These results suggest that habitual slips were not only more frequent than random errors but that this distinction became increasingly pronounced with more time to prepare a response—contrary to our prediction that longer response preparation time would mitigate habitual control.

Next, we examined whether response accuracy in the still-valued trials changed across time bins (*correct* ~ *binned response preparation time* + *(1 | ID)*). Participants were significantly less accurate in this shortest 0.2–0.5-s time window (*M* = 71.7, *SE* = 6.41) compared with the longer bins and significantly improved in the next time window (0.5–0.8 s), increasing by 7.61% (β = 7.61, *SE* = 1.89, *p* = 0.0001). Further improvements were observed in the 0.8–1.1-s bin (β = 7.98, *SE* = 1.89,* p* < 0.0001) and the 1.1–1.4-s bin (β = 9.75, SE = 1.89,* p* < 0.0001), reaching an average of 81.5% (SE = 6.41) in the longest preparation window. These findings confirm that participants’ ability to select goal-directed responses to valued outcomes improves robustly with increased response preparation time (Fig. 3a). Pairwise comparisons between later time bins revealed no significant differences (0.5–0.8 s vs. 0.8–1.1 s: *p* = 0.99; 0.5–0.8 s vs. 1.1–1.4 s: *p* = 0.89), suggesting that accuracy improvements plateau after approximately 500 ms of preparation time. Sensitivity analyses using an alternative time bin (0.3–0.6 s) reproduced this pattern of results for all the aforementioned analyses (see supplementary results, Table S2a-b).

Finally, we examined how the overall tendency to withhold changes over preparation time bins (*withheld* ~ *binned response preparation time* × *trial type* + *(1|ID))*. Across bins, withholding was substantially lower on valued vs devalued trials (main effect of trial type: β = − 62.23, *SE* = 7.91, *p* < 0.0001), and bin × trial-type interactions were not significant (all *p* > 0.59). In the shortest preparation window (0.2–0.5 s), participants withheld responses on approximately 83% of devalued trials (SE = 6.0). There were no significant changes in withholding from the 0.2–0.5 s to the 0.5–0.8-s bin (β = 1.45, *SE* = 1.51, *p* = 0.34), 0.8–1.1-s bin (β = –1.95, SE = 1.51, *p* = 0.20), or 1.1–1.4-s bin (β = –0.50, SE = 1.51, *p* = 0.74) (Fig. 3b). In the valued trials, 21.5% of responses in the shortest 0.2–0.5 s time bin consisted of withheld responses; however, unlike the devalued trials, we observed a significant decrease in withheld responses over time (0.2–0.5 s vs. 0.5–0.8 s: β = –4.35, *SE* = 1.67, *p* = 0.010; 0.2–0.5 s vs. 0.8–1.1 s: β = –4.89, *SE* = 1.67, *p* = 0.004; 0.2–0.5 s vs. 1.1–1.4 s: β = –5.94, *SE* = 1.67, *p* = 0.0006). Therefore, on both trial types, there was a general tendency to withhold responses at the short preparation time bin. To control for this more directly, we computed a per-participant Habit Index (*1 − (P[press|Valued] − P[press|Devalued]*) and modelled its change over preparation time bins (*Habit Index* ~ *binned response preparation time* + *(1|ID)*). We found that responding was consistently higher for valued than devalued outcomes across all time bins. Specifically, the habit index was positive at 0.2–0.5 s (model intercept: β = 0.378, *SE* = 0.104, *p* <0.0001), then decreased with additional preparation time: 0.2–0.5 s versus 0.5–0.8 s (β = − 0.0596, *SE* = 0.0186, *p* = 0.0019), marginally at 0.2–0.5 s versus 0.8–1.1 s (β = − 0.0334, *SE* = 0.0186, *p* = 0.0766), and decreased at 0.2–0.5 s versus 1.1–1.4 s (β = − 0.0558, *SE* = 0.0186, *p* = 0.0035; Fig. 3e). The decrease in habit index in the 0.8–1.1 s time bin became significant after excluding the two participants who were not clearly following the devaluation instructions (β = −0.042, SE = 0.019, p = 0.030; see supplementary results, Figure S8). Therefore, in direct contrast to examining raw responses to devalued outcomes, when controlling for valued responses, we found that with more preparation time, the habit index decreases.

### Remapping test results

In the Remapping experiment, participants were introduced to a revised S–R mapping (instead of Outcome Devaluation) after 6 days of training. We used a forced reaction time approach once again to systematically vary the preparation time participants had to generate their responses to the stimuli (Fig. 1b). We ran a linear mixed-effects model to examine the effect of preparation time bins on habitual responses (i.e., incorrect (old) responses to remapped trials) (*habit* ~ *binned response prep time* + *(1 | ID)*). In contrast to the devaluation experiment, as preparation time increased, habitual responses declined. In the 0.5–0.8-s time bin, participants showed a 19.43% reduction in habitual slips relative to the shortest time bin (β = − 19.43, *SE* = 3.71, *p* < 0.001). This downward trend continued in the later bins, with habitual responses decreasing by 26.74% in the 0.8–1.1 s window (β = − 26.74, *SE* = 3.71, *p* < 0.001) and by 27.81% in the 1.1–1.4 s window (β = − 27.81, *SE* = 3.71, *p* < 0.001; Fig. 3d).

We fit a linear mixed-effects model to examine how correct responses varied across original mapping vs. remapped trials, and whether this effect changed with preparation time (*Correct Response* ~ *Trial Type* × *binned preparation time* + *(1 | ID)*). In the shortest preparation time bin (0.2–0.5 s), participants were highly accurate in the original mapping trials (*M* (*SD*) = 88.79 (2.95)), while showing substantially less accuracy in remapped trials (*M* (*SD*) = 63.55 (3.97)), exhibiting significantly fewer correct responses in remapped trials compared with original trials under the highest level of time pressure (main effect of trial type: β = –25.24, *SE* = 3.66, *p* < 0.001). Response accuracy improved with longer preparation time across both consistent with original mapping and remapped trials, with correct responses increasing by 9.58% in the 0.5–0.8 s bin (β = 9.58, *SE* = 3.62, *p* = 0.009), 10.26% in the 0.8–1.1 s bin (β = 10.26, *SE* = 3.62, *p* = 0.005), and 10.41% in the 1.1–1.4 s bin (β = 10.41, *SE* = 3.62, *p* = 0.004) in the remapped trials. Importantly, the trial type × preparation time interaction revealed that the difference in accuracy between remapped and original trials narrowed with additional time. While the interaction at 0.5–0.8 s was marginally significant (β = 9.97, *SE* = 5.12, *p* = 0.053), it became significant in the later 0.8–1.1 s (β = 16.61, *SE* = 5.12, *p* = 0.001) and 1.1–1.4-s time bins (β = 17.52, *SE* = 5.12, *p* < 0.001), suggesting that the initial accuracy gap between remapped and original trials became progressively smaller as preparation time increased. These findings suggest that while remapped trials initially show lower accuracy, additional preparation time enables participants to partially overcome these habitual interference effects, with performance in remapped trials catching up to that of original mapping trials over time (Figs. 3c and d).

As with the devaluation task, we calculated a Habit Index for Remapping in a similar way = *1 − (P[correct press|Original mapping] − P[incorrect press|Remapped]*), which treats correct responses on original trials as a baseline accuracy measure and incorrect responses on remapped trials as habitual slips (i.e., failure to adapt the behaviour). We fit a mixed model (*Habit Index* ~ *binned response preparation time* + *(1|ID)*) and found the index was high at 0.2–0.5 s (β = 0.504, *SE* = 0.041, *p* < 0.0001) and then decreased sharply with preparation time: 0.5–0.8 s (β = − 0.319, *SE* = 0.0339, *p* < 0.0001), 0.8–1.1 s (β = − 0.391, *SE* = 0.0339, *p* < 0.0001), and 1.1–1.4 s (β = − 0.408, *SE* = 0.0339,* p* < 0.0001; Fig. 3f), indicating that habit expression falls sharply with more preparation time in the Remapping task.

### Direct comparison of habitual responses between tasks

To directly compare tasks in how response preparation relates to habitual slips, we used a linear mixed model (*habit* ~ *binned response preparation time × task* + *(1 | ID)*). Results revealed that habitual responses were significantly higher in the Remapping task compared to the Outcome Devaluation task at the shortest 0.2–0.5 s preparation time bin (main effect of task: β = 12.84, *SE* = 4.13,* p* = 0.002) (Fig. 3e). There was a significant interaction between task and preparation time at all subsequent time bins—0.2–0.5 vs. 0.5–0.8 s (β = − 11.55, *SE* = 4.51, *p* = 0.011), 0.2–0.5 vs. 0.8–1.1 s (β = − 16.49, *SE* = 4.51, *p* < 0.001), and 0.2–0.5 vs. 1.1–1.4 s (β = − 16.58, *SE* = 4.51, *p* < 0.001). As described above, this is due to habitual responding decreasing over time in the Remapping task, but increasing in the Outcome Devaluation task. Pairwise comparisons in individual bins showed that the difference between tasks was statistically significant only in the 0.2–0.5 s bin (*p* = 0.002) but not in later bins.

However, as stated above, this analysis does not control for baseline responding levels. We thus compared the Habit Index for each task over time (smaller habit index = fewer habit-consistent errors and more goal-directed responding; Outcome Devaluation Habit Index = *1 − (P[press|Valued] − P[press|Devalued]))*; Remapping Habit Index = *1 − (P[correct|Original mapping] − P[incorrect|Remapped]*; model = *Habit Index ~ task × time bin + (1|ID)*). Across tasks, there was an overall drop in the habit index with more preparation time (vs. 0.2–0.5 s: 0.5–0.8 s, β = − 0.0596, *SE* = 0.0271, *p* = 0.029; 0.8–1.1 s, β = − 0.0333, *SE* = 0.0271, *p* = 0.219; 1.1–1.4 s, β = − 0.0558, *SE* = 0.0271, *p* = 0.040; Figs. 3e & f), indicating fewer habitual slips as time increases. Critically, the task × time interactions showed larger decreases in Remapping relative to Devaluation (task × time 0.5–0.8 vs. 0.2–0.5: β = − 0.259, *SE* = 0.039, *p* < 0.0001; 0.8–1.1 vs. 0.2–0.5: β = − 0.357, *SE* = 0.039, *p* < 0.0001; 1.1–1.4 vs. 0.2–0.5: β = − 0.351, *SE* = 0.039, *p* < 0.0001), indicating that habitual slips diminish more rapidly with additional preparation time in Remapping task than in Outcome Devaluation.

### S–O associations explicit knowledge test

**Fig. 4:**
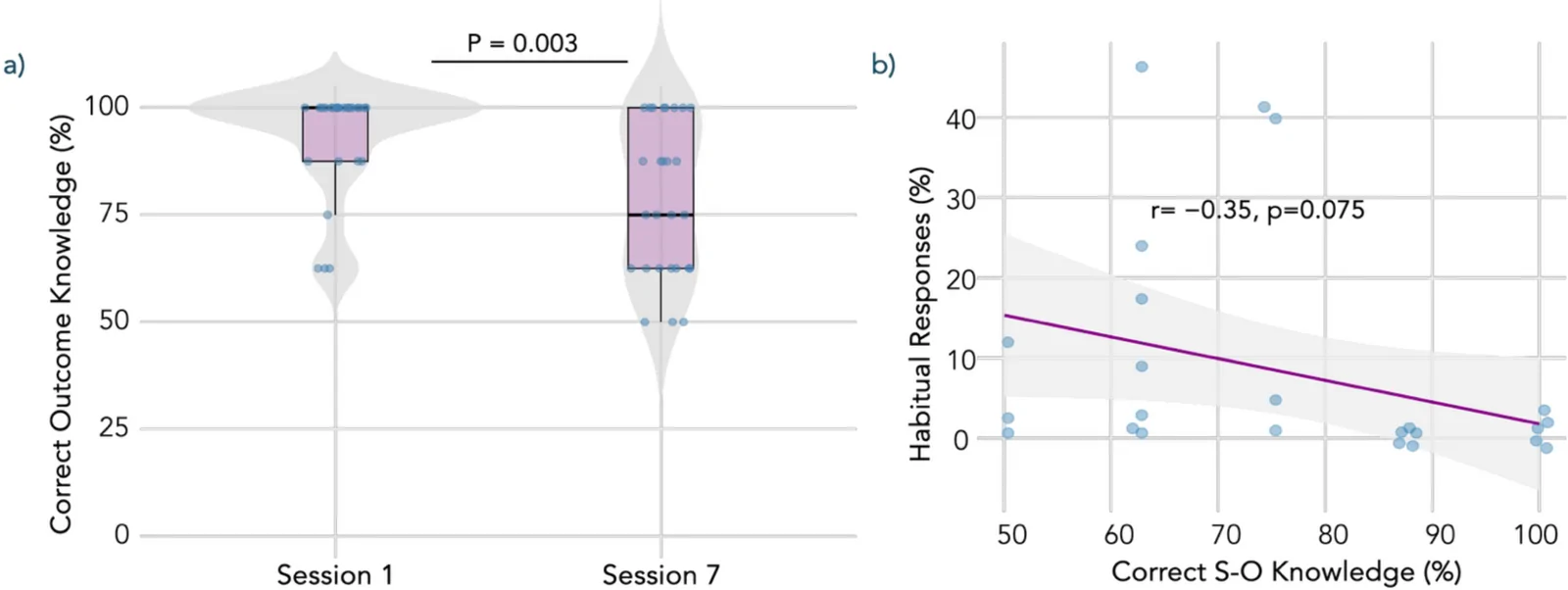
S–O Outcome knowledge. (**a**) Explicit outcome knowledge test comparison between the first and the final sessions shows a significant decrease in the outcome knowledge from sessions 1 to 7 (β = − 14.352, *SE* = 3.512, *p* = 0.0003). (**b**) The correlation between S–O knowledge test performance and average habitual responses in the outcome devaluation test across all time bins (r = − 0.35, *p* = 0.075)

In Experiment 1, participants completed an explicit knowledge test of S–O associations after the Pavlovian phase in Session 1 and once again, prior to the Outcome Devaluation test in Session 7. To ensure everyone had the required outcome knowledge necessary to perform the devaluation test, participants had to repeat the trials that were responded to incorrectly; the number of trials needed to complete this ranged between 8–19 trials (*M* (*SD*) = 9.32 (3.28). Prior to these repeats, these trials served as a test of outcome knowledge, thus testing the effectiveness of the manipulations. We ran a linear mixed effects model to investigate changes in S–O association knowledge from Session 1 to Session 7. The model (*percentage correct ∼ session* + *(1∣ID))* showed a statistically significant main effect of session corresponding to a decrease in S–O knowledge test from Session 1 (*M* (*SD*) = 93.05% (12.65)) to Session 7 (*M(SD)* = 78.7% (17.95)) (β = − 14.352, *SE* = 3.512, *p* = 0.0003) (Fig. 4a). An exploratory Pearson correlation analysis revealed a trend for negative association between knowledge test performance in Session 7 and habitual responses (*r* = − 0.35, *p* = 0.075, 95% CI = [− 0.64, 0.04]) (Fig. 4b). A significant negative association between Session 7 outcome knowledge and the mean Habit Index was revealed after excluding two participants who were not following devaluation instructions (r = −0.39, p = 0.042, 95% CI = [−0.66, −0.02]), indicating that participants with poorer explicit S–O knowledge showed less valued-over-devalued discrimination.

To explore whether time-pressured performance in the Outcome Devaluation task is related to explicit S–O knowledge, we linked session 7 outcome knowledge (% correct, 8 trials) to habit index score and modelled its change over preparation time bins (*habit index* ~ *preparation time bin* × *s–o knowledge* + *(1 | ID)*). Higher outcome knowledge showed a trend toward a smaller habit index (β = –0.024, *SE* = 0.020, *p* = 0.076), and time bin × knowledge interactions were not significant. This shows that participants who knew the S–O mappings better tended to show a larger valued-over-devalued response overall (i.e., fewer habitual slips). The main effect of knowledge became significant after excluding two participants who were not following the devaluation instructions (β = −0.0053, SE = 0.0024, p = 0.035), suggesting that participants with stronger S–O knowledge showed fewer action slips, directly consistent with the idea that devaluation-insensitive responding partly reflects inaccurate outcome representations. The nonsignificant time bin × S–O knowledge interactions indicate that knowledge did not change the time-course of the habitual response across preparation-time bins.

## Discussion

The present study investigated the expression of habitual behaviour using a widely used paradigm, Outcome Devaluation, and a more recently developed method, response Remapping, with identical multiple-session S–R–O training settings and tested under systematic variation in time constraints. When we indexed “habitual slips” directly from raw responses (i.e., responses to devalued outcomes in Outcome Devaluation; old-mapping errors in Remapping), the two paradigms strikingly showed different time-courses of habitual responses. In the Remapping experiment, we replicated previous findings that habitual responding decreased with increased preparation time (Hardwick et al., 2019; Martínez-López et al., 2025; Van Dessel et al., 2024). Habitual errors peaked around 200–500 ms after the stimulus was presented, and participants could partially overcome these habitual slips with more preparation time. This is consistent with the idea that habitual responses are fast and automatic but can be overridden by slower, deliberative control processes (Hardwick et al., 2019; Keramati et al., 2011; Luque et al., 2020; Moors & De Houwer, 2006). In the Outcome Devaluation paradigm, raw responses to devalued outcomes did not decrease with preparation time as expected and modestly increased in later time windows. After excluding the two participants who pressed against the devaluation instruction, this raw increase was substantially attenuated. The pattern on raw devalued presses is therefore not a robust signature of habit expression under time pressure and is more parsimoniously interpreted as goal-directed control operating over outcomes whose value some participants did not, in fact, update following the instructed devaluation. However, when we control for baseline response propensity (i.e., valued responding) in a Habit Index, we replicated the Remapping result. That is, the habit index decreased somewhat with more time, such that there were fewer habitual slips when more time was available. Because the Habit Index is defined on the difference between valued and devalued (or original and remapped) responding, it controls for overall accuracy and withholding within each time bin, indexing value‑ or mapping‑based responding rather than raw error counts. Importantly, though qualitatively similar, this effect was significantly smaller than for Remapping, showing that habit‑consistent responding attenuates much more steeply over time in the Remapping task than in Outcome Devaluation.

The Outcome Devaluation paradigm has long been criticised because of various limitations, including the overreliance on overt instruction, the low cost associated with habitual responding, reliance on null effects, and issues with the salience of the reinforcers and their devaluation (De Houwer et al., 2018; Gera et al., 2023). Most crucially, it has faced replication challenges in both humans (de Wit et al., 2018; Gera et al., 2023; Pool et al., 2022) and rodents (Bouton, 2019, 2021; Garr et al., 2020, 2021; Schreiner et al., 2020). Forced-response Remapping paradigms (Hardwick et al., 2019; Luque et al., 2020) have emerged as an appealing alternative, showing greater sensitivity to overtrained habitual tendencies, particularly in conditions that limit preparation time. However, even within Remapping studies, measures, such as RT switch costs and habitual error rates, have demonstrated limited convergent validity, suggesting they may tap into distinct facets of habit expression, such as habitual response preparation versus initiation (Du & Haith, 2025; Martínez-López et al., 2025; Michiels et al., 2025). Together, these divergences underscore that “habit” is not a unitary construct, but likely reflects multiple, dissociable processes depending on task demands. Notably, our Habit Index results show that in both paradigms, participants exhibit less habit with more time; yet the attenuation is markedly smaller in Outcome Devaluation than in Remapping.

Response Remapping likely captures automatic action selection under conflict, while devaluation tasks emphasise inhibitory control and outcome knowledge—each subject to different cognitive and motivational influences (Du & Haith, 2025; Du et al., 2022; Nebe et al., 2024). Indeed, a crucial difference may be that the Outcome Devaluation task requires participants to decide whether to respond at all, potentially encouraging a response withholding bias. Data from not-on-time trials also support this interpretation; very few late responses occurred on devalued trials, especially under the shortest preparation time bin. This suggests a two-stage decision-making process: first, deciding whether to respond at all, and then selecting an appropriate response (Du & Haith, 2025; Sjoerds et al., 2016). Two interpretations are compatible with this pattern: action slips may be masked at short preparation windows by an early withholding bias and emerge once that bias is overcome when sufficient time is available; alternatively, the late-bin responses on devalued trials may reflect goal-directed responding under an outcome representation that did not follow the devaluation instructions. Supporting this general withholding bias, even in valued trials, participants withheld a substantial number of responses compared to the Remapping task. Crucially, when we account for withholding in our Habit Index analysis, the pattern aligns across tasks: habitual slips decrease with additional time in both paradigms, and withheld responses in Outcome Devaluation reduce the observable signal of habit expression unless relative press propensities are explicitly contrasted. This logic is closely aligned with previous work that indexes devaluation sensitivity by comparing responses for still‑valued and devalued outcomes (de Wit et al., 2018; Hinojosa-Aguayo & González, 2020; Klossek et al., 2008), and here we show that, in a time‑pressured classical devaluation task with withholding, applying such contrast‑based metrics can reveal a modest decrease in habit over time that is otherwise obscured when looking at raw devalued responses alone.

 Our results show that participants with poorer explicit knowledge of S–O associations also showed a higher Habit Index, consistent with prior work (Gillan et al., 2011; Hennessy et al., 2025) and with the view that devaluation-insensitive responding partly reflects an inaccurate or unupdated outcome representation rather than purely S–R habit expression (De Houwer et al., 2018). This is a rather straightforward association to interpret: if one fails to learn what outcome follows a stimulus, then it is impossible to perform well at a devaluation test, regardless of how much time one has to respond. However, the effect is surprising in some ways, because after assessing explicit S–O knowledge, we explicitly retrained participants on S–O knowledge prior to the habit test. This suggests that while participants achieved 100% on this refresher test, their outcome knowledge remained weak. A study by De Houwer & colleagues (2018) found that knowledge about outcomes is poor in tasks of this sort, unless Outcome Devaluation tests specifically target explicit knowledge about those outcomes (De Houwer et al., 2018). For example, if participants primarily focused on learning which response earned points (rather than the specific outcome it led to), their responses could remain goal-directed, but they would nonetheless fail a devaluation test focused on specific outcomes that the experimenter deemed motivational. This issue has meant that apparent habitual errors might reflect poor outcome knowledge, limiting the interpretation of devaluation insensitivity as habitual responding (Buabang et al., 2021; De Houwer et al., 2018). We argue that many “slips” reflect goal‑directed control operating over an incorrect S–O knowledge, rather than purely S–R habits, in line with critiques of devaluation‑based habit measures (Buabang et al., 2021). Accordingly, we interpret these responses more cautiously as *habit‑consistent* or *devaluation‑insensitive* behaviour that may arise from a mixture of S–R and goal‑directed control, rather than as a pure readout of a dedicated “habit system” (De Houwer, 2019). We also observed that outcome knowledge significantly decreases throughout training, which adds an important piece of context to this finding. Reduced S–O knowledge may be a natural result of the habit formation process, where outcome representations become less relevant as training progresses, potentially contributing to habitual responding without fully determining it (van Timmeren & de Wit, 2023). This finding may also explain the observed withholding bias on devalued trials, as uncertainty due to reduced S–O knowledge could lead participants to withhold responses entirely (Watson et al., 2023).

Recent comparative work also shows that forced-response Remapping and Outcome devaluation are each reliable but not convergent, indicating they tap different control processes: Remapping indexes persistence of a trained S–R under reversal (i.e., response preparation conflict), whereas devaluation captures costs at initiation/inhibition when a response must be withheld against a learned tendency (Martínez-López et al., 2025). These distinctions fit with broader evidence that habits and goals can compete and interact over time, with preparation time dynamics shaping which governs each action (Wood et al., 2022). Understanding the distinct processes involved in habitual action selection (i.e., response preparation vs. initiation, as suggested by Du & Haith, 2025) may advance our understanding of when and why clinical interventions for compulsive disorders work. For example, approaches that emphasise response withholding (e.g., abstaining from smoking) may heavily tax cognitive control, making them challenging for populations with impaired executive functions (Horváth et al., 2022; van Timmeren et al., 2022). Alternatively, strategies targeting response substitution (e.g., replacing smoking with gum chewing), which involves forming a new association, can effectively overwrite old associations and offer potentially more viable routes for habit modification in clinical contexts (Gardner et al., 2021; Wood & Neal, 2007). Recognising these distinct habit forms provides a clearer theoretical framework for understanding habitual behaviour in real-world scenarios, potentially improving targeted interventions for habit-related psychopathologies, such as OCD, addiction, impulse control disorders, and so forth (Vandaele & Ahmed, 2021).

It is worth noting that our goal is not to claim that Outcome Devaluation and response Remapping tap different “types” of habit, but that, under identical training and timing constraints, they yield different behavioural readouts, because they operationalise control differently at test. Even with harmonised procedures, these distinct response requirements can plausibly produce divergent time courses. Crucially, what counts as “habit” also depends on how it is computed within a paradigm. For example, in the Outcome Devaluation task, looking only at raw presses on devalued trials in the full sample suggested a paradoxical increase with preparation time, but this pattern was largely carried by two participants whose responses were inconsistent with the devaluation instruction; once these were excluded, the raw-press time effect is reduced to a single time bin (0.8–1.1 s). The Habit Index, which subtracts devalued from valued press rates and thereby controls for overall withholding, decreases reliably across all three response preparation time bins, matching the direction seen in Remapping. We therefore interpret the contrast between these experiments as task and metric sensitivity to different facets of the goal-directed and habitual interplay, not as evidence for separate constructs of habit. A strong causal test will require future work that orthogonally manipulates mapping and response mode within-subjects (e.g., crossing devaluation with withholding vs remapping with press requirements), but the present design minimises procedural confounds and supports the claim that operationalisation (both task and the measure) meaningfully shapes the observed dynamics of behavioural control. Accordingly, our findings are best taken as evidence that common human “habit” tasks differ in what they measure and that, under our conditions, the Remapping task offers a more sensitive assay of habit‑consistent interference than the Outcome Devaluation paradigm. Our findings speak to the nonunitary measurement of habits: Response Remapping appears well‑suited to capturing fast time‑pressured expression of pretrained S–R links, whereas the Outcome Devaluation used here is heavily influenced by S–O knowledge, devaluation instructions, and withholding biases.

This study had some limitations. First, both tasks employed only two response options, limiting the ability to distinguish between habitual slips and random errors. Previous Remapping studies often used four S–R pairings (Hardwick et al., 2019), which reduces the probability of random errors aligning with trained responses. However, our task design was constrained by the structure of the Outcome Devaluation paradigm, where one outcome was devalued and the other remained valued, necessitating this simpler two-response format to maintain consistency across conditions. Second, the two experimental groups consisted of separate cohorts. Despite harmonised procedures and within-paradigm mixed-model inferences, between-task differences should be read cautiously; future studies should randomise participants to experiments (and include a minimal-training arm) or use within-subject crossover to establish convergent validity. Furthermore, our study did not manipulate training duration and compare its effects on habit expression (Hardwick et al., 2019). We opted instead for a single and significant amount of training in each group. Evidence shows that habit formation in simple laboratory-based behaviours can even occur within a single day, provided that a high number of repetitions (e.g., 1000 responses) are achieved (Garr et al., 2021); however, there is no gold standard in terms of the number of training sessions needed to train a habit. Nonetheless, the absence of a no-practice/minimal-training control group to anchor baseline choice probabilities under time pressure is a limitation of the current study. Without that arm, we cannot definitively separate early habit slips due to overtraining from any random errors at very short preparation times. A within-subject design that orthogonally manipulates training dose and includes a minimal-training baseline would provide the strongest causal test in future work. Finally, the task demands were relatively low, as indicated by high baseline accuracy rates in responses during training sessions. This ease of task execution may have further limited the emergence of habitual control, especially in the Outcome Devaluation paradigm (Watson & de Wit, 2018), where inhibiting responses is less effortful than selecting between competing alternatives. Future studies could address some of these shortcomings by adopting more sensitive outcome revaluation methods designed to better capture subtle variations in habit expression (Watson et al., 2023). A further limitation is that our devaluation manipulation was implemented purely by instruction, and we did not collect data to check if they had indeed devalued the outcome. In the animal studies literature, devaluation is typically verified by a consumption test (i.e., reduced intake of the devalued outcome following sensory-specific satiety or illness pairing); the human equivalent is post-instruction preference ratings or a forced choice between still-valued and devalued outcomes (Gillan et al., 2015). We collected only an S–O knowledge test, which confirms that participants remember which outcome each stimulus produces, but does not confirm that they updated the outcome’s current value. In our study, a small subset of participants showed devaluation‑insensitive responding at all preparation times despite passing the S–O knowledge criterion (Fig. 3e), suggesting that some “completely habitual” profiles may instead reflect a failure to adopt the devaluation goal under otherwise goal‑directed control. Future work should incorporate direct measures of devaluation efficacy to better distinguish genuine devaluation insensitivity from cases where the devaluation instruction is not understood or applied. But this said, it is important to flag this as a broader issue with devaluation tasks in general – if researchers must adopt ever-increasing interventions to ensure participants are aware of goals, it seems likely that these goals lack significance for the participants. Relatedly, our outcomes (faces and landscapes paired with points) are not primary reinforcers and may be too abstract, with no satiety analogue and no straightforward cost to attach to the devalued stimulus, making the participants less motivated to actually devalue the outcome. We make no claim that the Outcome Devaluation paradigm as implemented here taps the functioning of a dedicated habit system, and we adopt the more cautious interpretation that responses on devalued trials reflect a mixture of S–R habit expression and goal-directed control over outcome representations that may not have been updated by the instruction (Buabang et al., 2021; De Houwer et al., 2018).

## Conclusions

In conclusion, this study provides a direct comparison of habit expression between the classical Outcome Devaluation and response Remapping tests under time constraints and highlights that the expression of habitual control is highly dependent on task demands and operationalisation of habits. Outcome devaluation and response Remapping paradigms appear to tap into distinct aspects of habitual behaviour, response inhibition versus action selection, each interacting differently with response preparation time. These results emphasise that habit formation is not a uniform process and suggest that future research should take task-specific approaches into account to better capture the multifaceted nature of human habits.

## Supplementary Information

Below is the link to the electronic supplementary material.

Supplementary file1 (DOCX 1310 KB)

## Data Availability

All original data are publicly available online on the Open Science Framework at https://osf.io/yf394/?view_only=fd4774e3f6154e7aac7bb2e34f7f0a9e.

## Code availability

All R codes to recreate the main findings of this study and generate figures are available at https://osf.io/yf394. The Python and JavaScript codes, materials, and resources for building both PsychoPy and Pavlovia experiments are publicly available at: https://osf.io/yf394, https://gitlab.pavlovia.org/Parnianrafei/co_habitv5.git (Experiment 1), and https://gitlab.pavlovia.org/Parnianrafei/co_habit_remapv1.git (Experiment 2).
