# Supplementary material for: Does time pressure reveal habits? Divergent results from outcome devaluation and response remapping paradigms
Source: Cogn Affect Behav Neurosci. 2026 Aug 5;26(5):2018–34. doi: 10.3758/s13415-026-01474-6 (PMC13615044; doi:10.3758/s13415-026-01474-6)
Supplement: Supplementary file 1 [file 13415_2026_1474_MOESM1_ESM.docx]

**Supplementary materials**

**Does Time Pressure Reveal Habits?**

**Divergent Results from Outcome Devaluation and Response Remapping Paradigms**

**Parnian Rafei**^1,2^**, Steph F. Suddell**^1,2^**, Eike K. Buabang**^1,2^**, Gonzalo P. Urcelay**^3^**, Claire M. Gillan**^1,2^*****

1. Trinity College Institute of Neuroscience (TCIN), Trinity College Dublin, Dublin, Ireland
2. School of Psychology, Trinity College Dublin, Dublin, Ireland
3. School of Psychology, University of Nottingham, Nottingham, United Kingdom

**Methods (supplementary)**

**Additional Procedures**

**Self-report Measures**

Participants completed the following demographic form, mental health self-report measures and questionnaire in the first session of the study:

- Demographic data of sex, educational level, country of residence, native language, race, and employment status.
- A set of 20 items for the ‘Compulsivity and Intrusive Thought’ factor (Wise & Dolan, 2020), a transdiagnostic factor of psychopathology identified through self-report assessments in previous research and is shown to be associated with goal-directed deficits (Gillan et al., 2016).
- Vividness of Visual Imagery Questionnaire (VVIQ; Marks, 1973), consisting of 16 questions on which participants score between 1 and 5 according to how vividly they imagine a familiar person, a shop, the sky or a countryside scene, leading to scores between 16 and 80.

The questionnaires were hosted by Pavlovia Surveys (<https://pavlovia.org/>) and built in PsychoPy (version 2024.1.4) (Peirce, 2007).

**Audio Test and Attention Checks**

Upon signing up, participants were provided with a comprehensive digital information sheet, which provided general information about the study, including audio requirements. It was explicitly mentioned that they need a laptop or desktop computer to participate in this study. To enhance data quality, participants had to pass an audio test at the very beginning of each session to make sure they could hear the audio components of the tasks properly. The audio test consisted of a female voice reading out random numbers (3 numbers), and the participants had to type in the number that they heard. They were told before agreeing to participate that using Bluetooth headphones is strictly prohibited, given the audio latency (i.e., the short delay between the audio signal and the output), and they are only allowed to use computer speakers because of the millisecond response precision required for this study (Bridges et al., 2020). Even though it has been shown that Prolific research participants tend to pay enough attention and are highly engaged with assigned cognitive tasks (Albert & Smilek, 2023; Douglas et al., 2023) – to ensure high data quality– every session included multiple attention checks (e.g., questions in which participants were asked to type the exact words that appeared on the screen (blue, red, purple, pink), and also their age that was then matched with the age provided by the participant in the demographic form.

**Simulation training**

Participants had to do a mental simulation training task for the aim of another part of this study. This part of the task was repeated in every training session (sessions 1–6). Participants were asked to complete a set of 30 trials, equally distributed in 3 blocks of 10 trials, in which they should mentally imagine a word that appears on the screen for 5 seconds. The words presented during the control training trials were categorised into animals and furniture and presented randomly. Participants had to rate the “vividness” of their mental imageries (while receiving “Vivid simulations are clear, rich and detailed mental images.” as a definition) and the effort they exerted on a visual analogue scale between 0 to 10.

**Habit Test Procedures**

**Experiment 1: Outcome devaluation task**

One of the critical tests of habit expression is whether a behaviour is sensitive to outcome devaluation. For this reason, in experiment 1, sensitivity to outcome devaluation was determined in the test session under devalued and non-devalued conditions, where participants were informed that “Sam” is now bored of volunteering for the organisations that lead to a specific activity (i.e., ‘an outcome’, seeing landscapes or meeting people in a counterbalanced order) and to avoid losing points, they need to withhold their responses to the organisations (i.e., stimuli) that would show them the “devalued outcome”– the activity that Sam was bored of. The other outcome was still valued; hence, they had to withhold their responses for two organisations and actively respond to the other two still-valued ones. Furthermore, based on the findings of previous studies (Hardwick et al., 2019), we expected that the habitual system can select potential responses rapidly, while the goal-directed system requires longer processing times for withholding responses to devalued outcomes. We therefore employed a forced-response paradigm in this outcome devaluation test for limiting the time participants had to prepare their responses in order to prevent preparation of goal-directed responses and unmask habitual action slips and explore the time-dependent sensitivity of responses to outcome devaluation. We introduced the forced-response paradigm and the outcome devaluation manipulation on the final test session (session 7). First, the participants were informed that they need to prepare their responses at a certain time – three tones were played during each trial, and they must respond to the volunteering request of the organisation with the third tone. Participants were also instructed that the organisations send their requests at different times during the day (i.e., the stimulus time presentation differs from trial to trial) – sometimes the participants were presented with the stimulus soon after the trial began, hence had ample time to prepare their response and respond to the stimulus (i.e., long preparation time); whereas, in some other trials, the stimulus appeared right before the third beep tone, giving them little time to process the stimulus and choose the correct response (i.e., short preparation time). The third beep tone (i.e., the allowed response time window) was played 1400 ms after the trial onset, and the stimulus appeared at a random time between 200 to 1400 ms since the beginning of the trial.

Before starting the main habit test, participants had to complete a series of criterion-based force-response practice trials (60 trials in each block) with both outcomes being still valued and had to practice responding on time (i.e., with the third beep tone). After making each response to a request from an organisation, they received feedback on their timing: when the response keypress was recorded before the third tone the screen showed *"Too soon!"*, and if it was recorded after the third tone they saw *"Too late!"* Message on the screen. The participants were instructed to respond to all the trials on time where possible and regardless of accuracy; otherwise, they need to repeat another block of practice if they have <80% of on-time responses within a block. The participants could only reach the next stage of the experiment, which was the outcome devaluation test, if they have responded to 80% of the trials within the allowed response time window.

Upon reaching the >80% on-time responses at the practice stage, participants did an explicit knowledge test of the stimulus-outcome associations (similar to the same test they had done in session 1) to make sure that they knew the S–O associations correctly. The only difference between this test on the test session and the first training sessions was the repetition of a trial in case of responding incorrectly to it in the test session to reach 80% of correct responses. The explicit knowledge test consisted of 8 trials, two for each association, and the participants received feedback about their response accuracy, and they had to repeat the trial in case of a wrong response.

After completing the explicit knowledge test of S–O associations, the participants received instructions about the devaluation of one of the outcomes (i.e., face or landscape, counterbalanced across participants). They were instructed that in the last part of this game, Sam will continue the volunteering work, but there will be some changes in the rules – Sam is bored of seeing one of the outcomes (i.e., face or landscape, counterbalanced across participants), and they won’t get any happiness points from volunteering for those specific organisations associated with the devalued outcome anymore and will only lose 5 points by responding to them. So, they should skip those jobs (by not pressing the key, i.e., withholding their response) and don't respond when they receive a request from those organisations. Lastly, before starting the outcome devaluation trials, participants were informed that their points would be converted to real money and would be paid to them as a bonus payment (maximum of £4). They would only gain points in this round if the responses were both on time and accurate to organisations that Sam was not bored of (i.e., still valued), and also for not responding to the organisations that Sam doesn't want to volunteer for anymore (i.e., devalued). During this part of the experiment, participants were shown a question mark, instead of the outcome of their responses (i.e., faces or landscapes) upon making their choices on time. They did not receive feedback about their timing in this part of the experiment. The outcome devaluation test consisted of 360 trials (18 blocks of 20 trials), allowing the participants a response preparation time between 200–1400 ms, and the allowed response time was randomly distributed across trials. Participants could take a break between blocks of 20 trials.

The test session then ended with two questions asking the participants about their feedback on the game – a multiple-choice question about the difficulty of the outcome devaluation test asking *“How hard was it for you during the last part to NOT RESPOND to the organisation that Sam was bored of volunteering for?”* with the following response options: *'very easy', 'easy',' neutral', 'hard', 'very hard* (Figure S5a)'*,* and an open textbox for general feedback in which the participants could type in their comments about the study.

**Experiment 2: Remapping task**

The initial procedures of the remapping task and training sessions were identical to those used in the Outcome Devaluation task. Participants completed the same six days of training with consistent S–R–O contingencies, and on the final test day (Session 7), they first practised responding under time pressure using the forced-response paradigm. Only after reaching the timing criterion (80% on-time responses), they were introduced to the revised stimulus-response mappings unique to the remapping condition. To assess whether habitual responses override goal-directed flexibility when S–R associations are reversed, participants in Experiment 2 completed a response reversal (remapping) for the habit test. This test examined whether, after extensive training, participants would continue to prepare previously learned responses or flexibly update their behaviour in line with revised S–R mappings.

Similar to experiment 1, before beginning the remapping test, participants completed a series of criterion-based forced-response practice blocks (60 trials per block) to ensure they learn to time their responses under time pressure. Participants received immediate feedback: “Too soon!” if they responded before the third tone, or “Too late!” if they responded afterwards. Only those achieving at least 80% on-time responses within a block were permitted to proceed to the main habit test phase.

Upon reaching this criterion, participants were informed that two of the four organisations had changed their preferred method of communication, so Sam should try to learn to communicate with them based on the new preferences. The responses associated with those organisations (i.e., stimuli) would now require a different way of responding than during the training phase, whereas the other two mappings remained unchanged. Participants were instructed that they needed to learn the new mappings in the practice block at their own pace, and subsequently, respond accurately under time pressure in the main test.

Participants then completed a practice block of 24 trials (6 trials per stimulus) in which they could respond at their own pace to learn the new mappings. Accuracy feedback was provided after each trial, and participants were told to focus solely on learning the new S–R associations without concern for timing. If they failed to achieve at least 80% accuracy on this block, it was repeated until the criterion performance was met.

Following this remapping practice, participants moved on to the main test phase, which consisted of 360 trials (18 blocks of 20 trials). Similar to the Outcome Devaluation task, no outcome feedback was provided (responses resulted in a question mark on the screen), and no response-timing feedback was given. Participants were instructed to respond as accurately and on time as possible to earn points that would contribute to a monetary bonus (maximum £4), although point feedback was not shown during the test. The forced-response design and variable preparation times were maintained throughout, allowing us to examine how time pressure modulates the expression of previously learned versus updated S–R associations.

Participants could take breaks between blocks of 20 trials. The test concluded with two debriefing questions identical to those in Experiment 1: a difficulty rating (i.e., “How hard was it to respond to the organisations that changed their method of communication?”) (Figure S5a) and an open-ended feedback textbox to collect qualitative impressions of the task.

|  |  | **Experiment 1**  **Devaluation (N = 29)** | **Experiment 2**  **Remapping (N = 28)** |
| --- | --- | --- | --- |
|  | **Age, M (SD)** | 35.57 (6.66) | 37.93 (10.77) |
|  | **Gender** |  |  |
|  | Woman | 53.6% (15) | 56.7% (17) |
|  | Man | 46.4% (13) | 40.0% (12) |
|  | Non-binary | 0% (0) | 3.3% (1) |
|  | **English as First Language** |  |  |
|  | Yes | 92.9% (26) | 93.3% (28) |
|  | No / Second Language | 7.1% (2) | 6.7% (2) |
|  | **Ethnicity** |  |  |
|  | White | 75.0% (21) | 76.7% (23) |
|  | Asian | 10.7% (3) | 10.0% (3) |
|  | Black | 10.7% (3) | 6.7% (2) |
|  | Mixed | 3.57% (1) | 3.3% (1) |
|  | Other | 0% (0) | 3.3% (1) |
|  | **Education** |  |  |
| Undergraduate Degree | | 64.3% (18) | 36.7% (11) |
|  | Further Education (A-levels) | 14.3% (4) | 33.3% (10) |
|  | GCSE or Equivalent | 14.3% (4) | 16.7% (5) |
|  | Postgraduate Degree | 7.1% (2) | 13.3% (4) |

**Supplementary Table 1. Demographic information of participants in experiment 1 (N=29) and experiment 2 (N=28).** Ethnic groups based on the 2021 Census of England and Wales ([ref](https://www.ethnicity-facts-figures.service.gov.uk/style-guide/ethnic-groups/)): White (English, Welsh, Scottish, Northern Irish or British, Irish, Gypsy or Irish Traveller, Roma, any other White background), Asian or Asian British (Indian, Pakistani, Bangladeshi, Chinese, any other Asian background), Black, Black British, Caribbean or African (any other Black, Black British, or Caribbean background), Mixed or multiple ethnic groups. Education levels were described as: Primary education, General Certificate of Education (GCSE or equivalent), Further Education (A levels or equivalent), Undergraduate Degree (Bachelor’s degree or equivalent), Postgraduate Degree (Master’s or Doctorate degree or equivalent)

**Results (supplementary)**

**Figure S3. Test results for outcome devaluation and remapping tasks (all responses without considering time constraints). (**a) Proportion of correct, incorrect, and withheld responses (%) to the still-valued stimuli in the outcome devaluation test, (b) Proportion of habitual, incorrect, and withheld responses (%) to the devalued stimuli in the outcome devaluation test, (c) Proportion of correct and incorrect responses (%) to the original mapping trials in the remapping test, (d) Proportion of correct and habitual responses (%) to the remapped trials in the remapping test.

**Figure S4. Proportion of early (<0.2 s) vs. late (>1.4 s) not-on-time responses.**
(a) In the devaluation task, participants made 12.4 % early and 87.6 % late presses on still-valued trials, and 13.8 % early and 86.2 % late presses on devalued trials.
(b) In the remapping task, there were 20.5 % early responses for Original mapping trials and 13.8 % in the Remapped, as well as late responses of 79.5 % and 86.2 % for each condition, respectively. Bars show group-level percentages for each condition.

**Forced-response blocks to criterion**

In order to assess how many forced-response criterion blocks it took for participants to reach 80% on-time responses, we calculated the number of blocks required to reach the learning criterion of having 80% of trials performed within the time window (i.e., when the third beep tone is played ± 100 ms tolerance). The results for Experiment 1 showed that the number of blocks the participants needed to reach 80% of on time responses ranged between 1–4 blocks (Mean (SD) = 1.76(0.78)). In Experiment 2, the number of blocks the participants needed to reach 80% of on time responses ranged between 1–6 blocks (Mean (SD) = 1.86(1.27)). We then compared the number of blocks required to reach the threshold of 80% on time responses between experiment 1 and experiment 2. The results indicated that participants in both tasks varied in the number of criterion blocks required (Figure S5a). A two-sample t-test was performed to compare the number of criterion blocks between the two tasks. The t-test revealed no significant difference between the two tasks (t(46.62) = 0.372, p = 0.711, 95% CI [-0.4559, 0.6628). These results suggest that the number of criterion blocks required to reach 80% on-time was similar across both tasks.

**Figure S5. forced-response criterion blocks to threshold and self-report level of task difficulty.** (a) the number of criterion blocks required to reach 80% on-time responses before beginning the habit test across both experiments (b) self-report level of difficulty of each habit test measured at the end of each experiment

**Sensitivity analysis with 0.3-0.6 as the reference time bin**

Our forced-response schedule constrained preparation time to 200–1400 ms. Unlike designs that also sample 0–200 ms (e.g., 4-choice remapping in Hardwick et al., 2019), we therefore do not observe the classic very-early “peak” in habitual slips; instead, accuracy rises steeply and asymptotes by ~600 ms in our results. In this sensitivity analysis, we re-estimate all models using 0.3–0.6 s as the reference bin, to ensure our effects are not an artefact of the original 0.2–0.5 s bin. This analysis excludes trials cued to respond at 200–300 ms post-stimulus since the shortest time bin starts from 300 ms. At the long end, our task did not record responses beyond 1400 ms, so the nominal 1.2–1.5 s bin contains trials only up to 1.4 s. Results and tables below use this alternative binning throughout.

For the Outcome Devaluation task, re-estimating the devaluation models with a stricter “early” window (0.3–0.6 s) yielded the same qualitative pattern of results as the bins in the original manuscript (i.e., 0.2–0.5 s). First, on devalued trials the dominance of previously trained (habitual) keypresses over other errors (Correct–Incorrect) increases with preparation time: β= 3.89, SE = 1.82, p = 0.036 at 0.6–0.9 s, β= 4.25, SE=1.82, p =0.022 at 0.9–1.2 s, and β= 3.79, SE=1.82, p = 0.041 at 1.2–1.5 s (Table S2A). Second, withholding on devalued trials remains high and stable (intercept = 85.1, SE = 5.75), with small, non-significant changes across time bins (e.g., β=−2.86 at 0.9–1.2 s, p = 0.057). Third, valued trials show the expected accuracy gains with time: β= 3.63 (SE=1.37, p = 0.0095) at 0.6–0.9 s, β= 3.40 (SE =1.37, p = 0.0149) at 0.9–1.2 s, and β= 6.08 (SE =1.37, p < .0001) at 1.2–1.5 s (Table S2B), mirrored by larger Correct–Incorrect differences. Even the raw habitual-keypress percentage on devalued trials remains higher than the 0.3–0.6 s reference in later bins (e.g., β= 3.55 at 0.9–1.2 s, p = 0.024). Although the 0.3–0.6 s reference time bin is a bit later (and thus more conservative) than 0.2–0.5 s, all three patterns in the findings reported in the manuscript persist: (i) valued accuracy improves, (ii) devalued withholding stays high, and (iii) habitual slips increasingly dominate other errors as time grows.

For the remapping task, re-binning to 0.3–0.6 s reference reproduces all substantive conclusions from our original analysis. Habitual slips on remapped trials decline sharply as preparation time increases (e.g., β= −16.9 to −21.7 across later bins, all p<0.001), while correct responses on remapped trials increase strongly (β= 16.9 to 21.7, all p<0.001). Accuracy on original-mapping trials (already high) shows small, reliable gains (β=3.7 to 4.9, all p<0.001). Critically, the Trial Type × time interaction remains robust (β= 13.4 to 17.0, p<0.0038; table S2E), indicating that the initial performance gap between remapped and original trials closes with additional preparation time. Thus, when re-analysing the remapping task responses, the same pattern holds when anchoring the model at 0.3–0.6 s, aligning with results reported in the original manuscript (reference bin: 0.2–0.5 s) and strengthening the robustness of our conclusions.

**Table S2a. Experiment 1: Devalued trials**

(Outcome: percentages at the ID×bin level; fixed effects are changes vs the reference bin)

| **Model & Contrast (vs ref)** | **Old bins β (ref 0.2–0.5 s)** | **New bins β (ref 0.3–0.6 s)** |
| --- | --- | --- |
| Correct response to devalued (i.e., habitual slip %) 0.5–0.8 (→ 0.6–0.9) | 3.81 (SE 1.96), p=0.056 | 2.44 (SE 1.55), p=0.119 |
| 0.8–1.1 (→ 0.9–1.2) | 6.56 (1.96), p=0.001 | 3.55 (1.55), p=0.024 |
| 1.1–1.4 (→ 1.2–1.5) | 5.57 (1.96), p=0.006 | 2.63 (1.55), p=0.093 |
| Correct − Incorrect (dominance of habitual over “other” errors) 0.5–0.8 (→ 0.6–0.9) | 9.06 (3.11), p=0.005 | 3.89 (1.82), p=0.036 |
| 0.8–1.1 (→ 0.9–1.2) | 11.18 (3.11), p=0.0004 | 4.25 (1.82), p=0.022 |
| 1.1–1.4 (→ 1.2–1.5) | 10.65 (3.11), p=0.001 | 3.79 (1.82), p=0.041 |
| Withheld (withhold % on devalued) 0.5–0.8 (→ 0.6–0.9) | 1.45 (1.51), p=0.34 | −0.99 (1.48), p=0.507 |
| 0.8–1.1 (→ 0.9–1.2) | −1.95 (1.51), p=0.20 | −2.86 (1.48), p=0.057 |
| 1.1–1.4 (→ 1.2–1.5) | −0.50 (1.51), p=0.74 | −1.47 (1.48), p=0.323 |

**Table S2b. Experiment 1: Valued trials**

(Outcome: percentages at the ID×bin level; fixed effects are changes vs the reference bin)

| **Model & Contrast (vs ref)** | **Old bins β (ref 0.2–0.5 s)** | **New bins β (ref 0.3–0.6 s)** |
| --- | --- | --- |
| Correct (valued accuracy %) 0.5–0.8 (→ 0.6–0.9) | 7.61 (1.89), p=0.0001 | 3.63 (1.37), p=0.0095 |
| 0.8–1.1 (→ 0.9–1.2) | 7.98 (1.89), p<0.0001 | 3.40 (1.37), p=0.0149 |
| 1.1–1.4 (→ 1.2–1.5) | 9.75 (1.89), p<0.0001 | 6.08 (1.37), p<0.0001 |

**Table S2c. Experiment 2: Remapped trials, habitual slips**

Model: Prop_Habit ~ binned time + (1|ID)
Reference bin: 0.3–0.6 s (new) vs 0.2–0.5 s (old, original manuscript)

| **Model & Contrast (vs ref)** | **Old bins β (ref 0.2–0.5 s)** | **New bins β (ref 0.3–0.6 s)** |
| --- | --- | --- |
| 0.5–0.8 (→ 0.6–0.9) | −19.43 (SE= 3.71), p<0.001 | −16.93 (SE= 2.66), p<0.001 |
| 0.8–1.1 (→ 0.9–1.2) | −26.74 (SE= 3.71), p<0.001 | −20.58 (SE= 2.66), p<0.001 |
| 1.1–1.4 (→ 1.2–1.5) | −27.81 (SE= 3.71), p<0.001 | −21.72 (SE= 2.66), p<0.001 |

**Table S2d. Experiment 2: Remapped trials, correct responses**

| **Model & Contrast (vs ref)** | **Old bins β (ref 0.2–0.5 s)** | **New bins β (ref 0.3–0.6 s)** |
| --- | --- | --- |
| 0.5–0.8 (→ 0.6–0.9) | 9.58 (SE= 3.62), p=0.009 | 16.93 (SE= 2.66), p<0.001 |
| 0.8–1.1 (→ 0.9–1.2) | 10.26 (SE= 3.62), p=0.005 | 20.58 (SE= 2.66), p<0.001 |
| 1.1–1.4 (→ 1.2–1.5) | 10.41 (SE= 3.62), p=0.004 | 21.72 (SE= 2.66), p<0.001 |

**Table S2e. Experiment 2: Trial Type (Remapped vs Original × time)**

Correct ~ Trial Type × binned preparation time + (1 | ID)

| **Model & Contrast (vs Original @ ref)** | **Old bins β (ref 0.2–0.5 s)** | **New bins β (ref 0.3–0.6 s)** |
| --- | --- | --- |
| Main effect: Remapped @ ref time | −25.24 (SE= 3.66), p<0.001 | −25.17 (SE= 3.25), p<0.001 |
| Remapped × 0.5–0.8 (→ 0.6–0.9) | 9.97 (SE= 5.12), p=0.053 | 13.38 (SE= 4.57), p=0.0038 |
| Remapped × 0.8–1.1 (→ 0.9–1.2) | 16.61 (SE= 5.12), p=0.001 | 16.97 (SE= 4.57), p=0.00027 |
| Remapped × 1.1–1.4 (→ 1.2–1.5) | 17.52 (SE= 5.12), p<0.001 | 17.02 (SE= 4.57), p=0.00026 |

Regarding the between-task comparisons, we re-ran the cross-task habit analysis with a reference window of 0.3–0.6 s (rather than 0.2–0.5 s) based on previous studies (Hardwick et al., 2019), using the same mixed model (Habit ~ task × bin + (1|ID)). The early-bin task difference remains significant and in the same direction: Remapping > Devaluation (β= 16.93, SE = 6.81, t = 2.49, p = 0.015). As in the original manuscript, later bins show no reliable between-task differences (0.6–0.9: β= −2.41, p = 0.724; β= 0.9–1.2: β= −7.19, p = 0.295; 1.2–1.5: β= −7.40, p = 0.280; Figure S6). The task × time bin interactions also confirm that the remapping–devaluation gap shrinks with time (task×bin: β= −19.35 to β=−24.34, all p < 0.001; Figure S6). In other words, the interpretive pattern reported in the manuscript (i.e., stronger early habit in Remapping that dissipates with more preparation time, versus a relatively stable profile in Devaluation) replicates when the reference is shifted to 0.3–0.6 s. Thus, the novel cross-task contrast does not depend on the initial (0.2–0.5 s) reference choice.

**Figure S6.** Between-task comparison of habitual responses with 0.3–0.6 s as the reference time bin

**Sensitivity analysis with exclusion of non-compliant participants**

Two further participants were excluded from the primary analysis post hoc because their Habit Index (1 − (P[press|Valued] − P[press|Devalued])) sat at ≈ 2 in every preparation time bin, indicating that they pressed against the devaluation instruction (i.e., pressed on essentially every devalued trial and withheld on essentially every valued trial). The results of the analysis with these participants removed (N=28) are reported as follows. In the full sample (N=30), we found that there was an increase in correct responses to devalued outcomes (i.e., pre-trained habitual slips) over time in two of the bins: β(SE) =5.87(1.86) at 0.8–1.1 s (p = 0.002) and β(SE) = 4.99(1.86) at 1.1–1.4 s (p = 0.009) relative to the first bin. However, after excluding those two ‘reverse’ participants, only the 0.8–1.1 s time bin remains significant (β = 3.41, SE = 1.44, p = 0.020) while the 1.1–1.4 s contrast loses statistical significance (β = 2.37, SE = 1.44, p = 0.103; Figure S7).

**Figure S7.** Outcome devaluation test results in devalued trials after excluding N=2 reverse responders.

Crucially, the Habit Index decreases significantly with more time across all three bin contrasts (β = −0.070, SE = 0.019, p = 0.0004 at 0.5–0.8 s; β = −0.042, SE = 0.019, p = 0.030 at 0.8–1.1 s (this was p = 0.077 in the full sample); β = −0.067, SE = 0.019, p = 0.0007 at 1.1–1.4 s; Figure S8) after excluding those two participants. Thus, this effect becomes slightly stronger after excluding those two participants with a Habit Index≈2 (i.e., reverse responders).

**Figure S8.** Habit Index changes across preparation time bins after excluding N=2 reverse responders.

**References**

Albert, D. A., & Smilek, D. (2023). Comparing attentional disengagement between Prolific and MTurk samples. *Scientific Reports*, *13*(1), 20574. https://doi.org/10.1038/s41598-023-46048-5

Bridges, D., Pitiot, A., MacAskill, M. R., & Peirce, J. W. (2020). The timing mega-study: Comparing a range of experiment generators, both lab-based and online. *PeerJ*, *8*, e9414. https://doi.org/10.7717/peerj.9414

Douglas, B. D., Ewell, P. J., & Brauer, M. (2023). Data quality in online human-subjects research: Comparisons between MTurk, Prolific, CloudResearch, Qualtrics, and SONA. *PLOS ONE*, *18*(3), e0279720. https://doi.org/10.1371/journal.pone.0279720

Gillan, C. M., Kosinski, M., Whelan, R., Phelps, E. A., & Daw, N. D. (2016). Characterizing a psychiatric symptom dimension related to deficits in goal-directed control. *eLife*, *5*, e11305. https://doi.org/10.7554/eLife.11305

Hardwick, R. M., Forrence, A. D., Krakauer, J. W., & Haith, A. M. (2019). Time-dependent competition between goal-directed and habitual response preparation. *Nature Human Behaviour*, *3*(12), 1252–1262. https://doi.org/10.1038/s41562-019-0725-0

Marks, D. F. (1973). Visual Imagery Differences in the Recall of Pictures. *British Journal of Psychology*, *64*(1), 17–24. https://doi.org/10.1111/j.2044-8295.1973.tb01322.x

Peirce, J. W. (2007). PsychoPy—Psychophysics software in Python. *Journal of Neuroscience Methods*, *162*(1), 8–13. https://doi.org/10.1016/j.jneumeth.2006.11.017

Wise, T., & Dolan, R. J. (2020). Associations between aversive learning processes and transdiagnostic psychiatric symptoms in a general population sample. *Nature Communications*, *11*(1), 4179. https://doi.org/10.1038/s41467-020-17977-w
